# Psychiatric Profiles of eHealth Users Evaluated Using Data Mining Techniques: Cohort Study

**DOI:** 10.2196/17116

**Published:** 2021-01-20

**Authors:** Jorge Lopez-Castroman, Diana Abad-Tortosa, Aurora Cobo Aguilera, Philippe Courtet, Maria Luisa Barrigón, Antonio Artés, Enrique Baca-García

**Affiliations:** 1 Institute of Functional Genomics, CNRS-INSERM Montpellier France; 2 Department of Psychiatry Nimes University Hospital Nimes France; 3 CIBERSAM Madrid Spain; 4 University of Montpellier Montpellier France; 5 Department of Signal Theory Universidad Carlos III de Madrid Madrid Spain; 6 Instituto de Investigación Sanitaria Gregorio Marañon Madrid Spain; 7 Department of Psychiatric Emergency and Acute Care Lapeyronie Hospital University of Montpellier Montpellier France; 8 Universidad Autonoma de Madrid Madrid Spain; 9 Department of Psychiatry Hospital Universitario Fundación Jiménez Díaz Madrid Spain; 10 Department of Psychiatry University Hospital Villalba Villalba, Madrid Spain; 11 Department of Psychiatry University Hospital Infanta Elena Valdemoro, Madrid Spain; 12 Department of Psychiatry University Hospital Rey Juan Carlos Mostoles, Madrid Spain; 13 Universidad Católica del Maule Talca Chile

**Keywords:** mental disorders, suicide prevention, suicidal ideation, data mining, digital phenotyping

## Abstract

**Background:**

New technologies are changing access to medical records and the relationship between physicians and patients. Professionals can now use e-mental health tools to provide prompt and personalized responses to patients with mental illness. However, there is a lack of knowledge about the digital phenotypes of patients who use e-mental health apps.

**Objective:**

This study aimed to reveal the profiles of users of a mental health app through machine learning techniques.

**Methods:**

We applied a nonparametric model, the Sparse Poisson Factorization Model, to discover latent features in the response patterns of 2254 psychiatric outpatients to a short self-assessment on general health. The assessment was completed through a mental health app after the first login.

**Results:**

The results showed the following four different profiles of patients: (1) all patients had feelings of worthlessness, aggressiveness, and suicidal ideas; (2) one in four reported low energy and difficulties to cope with problems; (3) less than a quarter described depressive symptoms with extremely high scores in suicidal thoughts and aggressiveness; and (4) a small number, possibly with the most severe conditions, reported a combination of all these features.

**Conclusions:**

User profiles did not overlap with clinician-made diagnoses. Since each profile seems to be associated with a different level of severity, the profiles could be useful for the prediction of behavioral risks among users of e-mental health apps.

## Introduction

The development of new technologies shows promise for causing a revolution in the way chronic diseases are followed and treated [1]. In the past few decades, we have seen the following two major technological changes directly connected to the availability of medical information: (1) introduction of electronic health records in most health care facilities, and (2) accessibility to portable devices capable of acquiring information about their users. Both systems are already being used to enhance communication between health providers and final users and to improve the overall performance of health care. Indeed, public and private entities are massively investing in the development of web-based platforms or smartphone apps through which patients can organize their medical agenda, have access to all or part of their medical records, provide their input, and join their medical referents [2].

It seems reasonable to believe that the follow-up of persons with mental illness will be improved if eHealth systems lead to an increased interaction with health care providers. Our group and others have shown that electronic assessment is feasible with proper adaptation [3]. Efficient monitoring may prompt health responses in cases of emergency [4], inform accurately about real-life behaviors between medical appointments, reduce unnecessary visits, and sustain therapeutic decisions [5]. Other parameters, such as biomarkers and input from close relatives, can be added to the monitoring system. This kind of ecosystem already exists [6], and a growing body of evidence has shown that eHealth tools improve treatment outcomes in terms of engagement, symptom improvement, well-being, and self-care [7-13]. Their combination with machine learning techniques has also shown positive impacts on the diagnosis, prediction, and prevention of several diseases, such as cancer [14-16].

Despite these advances, the majority of potential users, such as elderly persons with low educational levels [17], seem to be unenthusiastic about e-mental health tools [18]. Utilization rates have been associated with the characteristics of health professionals [19], but there is little knowledge about the kinds of patients who use e-mental health, how they become users, and what are their patterns of use. Young age, high education, and dissatisfaction with the health care system might be common features among eHealth users [20]. We do not know if this profile also applies to patients with mental disorders, but their digital phenotype [21] is likely to contain valuable information for clinicians and providers alike [22,23]. Alterations in the patterns of use could help clinicians to detect pathological or risky behaviors and individual needs, and increase treatment efficiency.

In this article, a nonparametric latent feature model based on the Indian Buffet Process (IBP) explores the response patterns of 2254 psychiatric outpatients to a web-based questionnaire. We aimed to define specific profiles according to response patterns and then link profiles with psychiatric disorders. The study will thus specifically describe patients with psychiatric diagnoses who have used an eHealth application at least once. We hypothesize that data mining techniques, such as IBP, can be used to associate information from different questionnaires and assessments in a plausible model that could serve ultimately to plan health care delivery.

## Methods

### Sample

Participants were recruited from psychiatric outpatient facilities in the catchment area of Fundacion Jimenez Diaz, a University Hospital in Madrid, Spain. This hospital is part of the National Health Service and provides medical coverage to about 850,000 people. From May 2014 onwards, all clinicians working at the six mental health centers of the catchment area received specific training and were encouraged to use the MEmind Wellness Tracker systematically in their clinical activity. A total of 2254 patients signed up on the MEmind platform and completed the assessment, and they were subsequently included in the study. The assessment comprised the collection of information about sociodemographic features and diagnoses. Participants also filled up a short questionnaire. For this study, we used broad inclusion criteria. Every patient attending psychiatric consultations independent of diagnosis was considered. Thus, all clinicians in the catchment area were instructed to propose the use of the web application to every outpatient they saw with no restriction whatsoever regarding their diagnoses or their clinical statuses. The total number of outpatients who consulted during the study period was 30,808.

For the purpose of this study, we included only participants who voluntarily accessed the application and responded to an open-text field. We made this choice to select proactive participants who completed most of the questions at the user end. We noted a missing data rate of 12%, which resulted from the sum of clinical missing data and a lack of completeness of the questionnaires at the user end of the application.

This study was performed in agreement with the ethic requirements of the Declaration of Helsinki (World Medical Association, 2013) and was approved by the Institutional Review Board of the University Hospital Fundación Jiménez Díaz (Madrid, Spain). All participants provided written informed consent to participate in the study.

### Assessment

#### Questionnaires

The data set consists of 23 questions from the following three different questionnaires: (1) a brief day assessment related to sleep quality, appetite, medication intake, aggressiveness, and suicidal behavior (six items); (2) the Who-5 Well-Being Index [24] (five items); and (3) the ninth version of the General Health Questionnaire [25] (12 items). All these questionnaires are short self-reported measures of current mental well-being. All items are yes-or-no questions, followed by the degree of agreement reported on a Likert scale (0 to 100 points). Although participants could repeat the assessment, only data from the first report were included in the model.

#### Clinical Diagnoses

Diagnostic coding was based on the International Statistical Classification of Diseases and Related Health Problems (ICD-10) [26]. Thus, diagnoses of mental disorders were classified into 10 groups (F0 to F9) according to ICD-10 (Multimedia Appendix 1). The corresponding physician coded the diagnosis for each patient and completed the clinical global impression (CGI) scale [27], which reflects the global functioning of a patient according to the view of the clinician on a scale (0 to 7 points). The CGI scale provides a summary measure accounting for patient history, psychosocial factors, behavior, and the impact of symptoms on the patient’s ability to function (Multimedia Appendix 2).

### Data Processing

**Figure 1:**
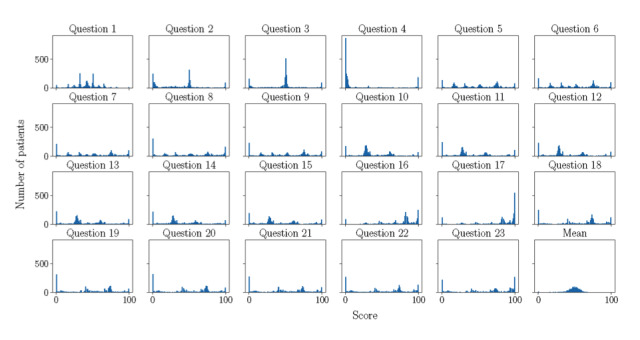
Histograms of scores provided by eHealth users to each of the 23 questions of the mental well-being questionnaire. Scores range from 0 to 100. The last histogram presents the average score for all questions.

First, the scores for the items with a positive valence in the questionnaire data set (items 1 to 15) were inversed. In this way, a higher score for any item of the questionnaire indicated poorer mental health. Second, we dichotomized every item score using a specific threshold in order to code the top 10% scores with the value “1” and the remaining 90% with the value “0” (Multimedia Appendix 3). The use of a centesimal scale increases the sensibility of the questionnaire. Responders tend to avoid extreme values unless they identify completely with them [28], but extreme responders do not seem to be affected by the length of the response scale [29]. By using the highest scores, we made sure that only the extreme responders were separated. The histograms of dichotomized scores for the items are shown in Figure 1.

The clinical records of the participants provided a second source of data. These records included sex, age, clinical diagnoses, and CGI values. CGI values presented missing data, so subsequent analyses including this variable were carried out with a total sample of 2000 participants. For analyses involving clinical diagnoses, the total sample was 1787 participants. All patients with missing data were excluded from this part of the data modeling. Comorbid diagnoses were also examined when present.

### Data Modeling

We applied the *Sparse Poisson Factorization Model* (SPFM) to model the data. The SPFM is based on the IBP [30], a nonparametric probabilistic method that proposes a sparse analysis of the variables. The input data for the model must be binary or categorical. The SPFM decomposes the input matrix into the following two nonnegative and disperse matrixes: matrix Z and matrix β. Latent factor sets can be calculated from them. The binary Z matrix represents the number of active factor sets for each patient (Multimedia Appendix 4). The β matrix weights the contribution of each factor set to each item of the questionnaire. Each factor set is characterized by precise values on the 23 questions. A higher weight (β) of a factor set for an item is associated with a greater probability to find a high score in that item when that particular factor set is active. The SPFM also estimates a *bias term*, a factor set that is present in all the patients of the sample [31]. The bias term is the “default” situation of an eHealth user and represents a profile shared by all patients that is independent of any additional feature. In that sense, the bias term allows the machine learning function to be shifted to better fit the data in a similar way as done by the y-intercept in an equation.

**Figure 2:**
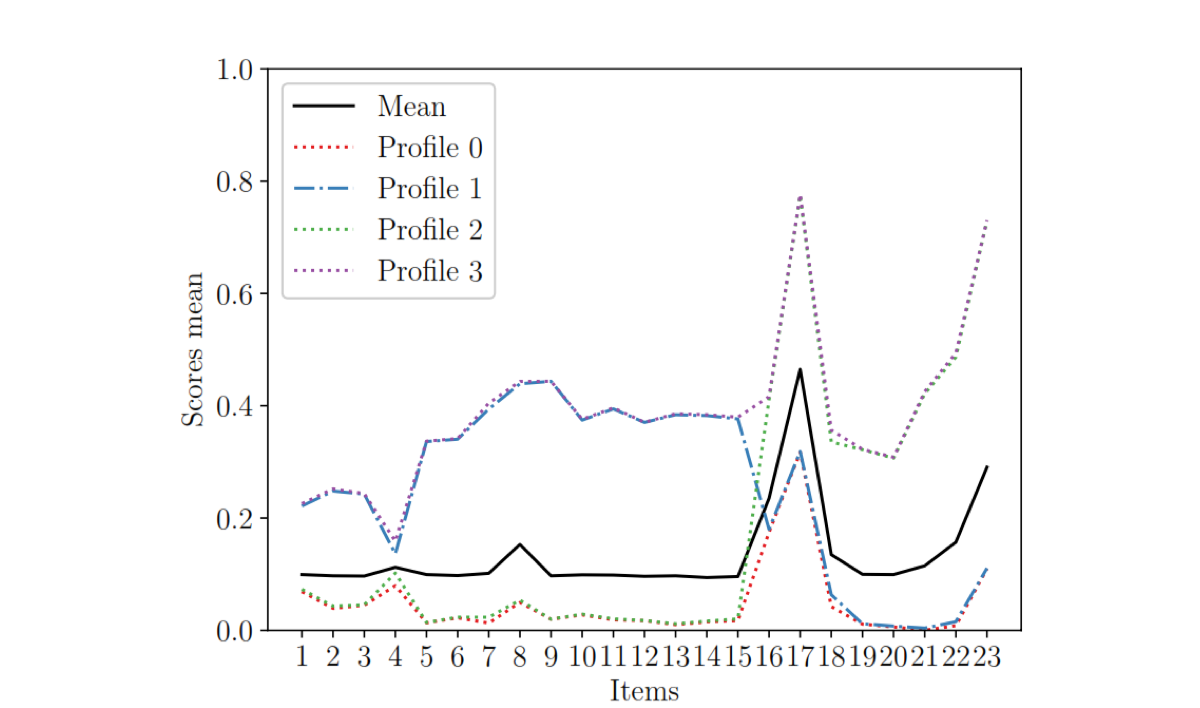
Item scores according to the four patient profiles and their averages for the whole sample.

Different profiles have been obtained by applying the basic clustering method K-means [32]. The procedure classifies a given data set through a certain number of clusters fixed a priori. This method, applied on the Z matrix, associates data with similar characteristics into different clusters by using centroids. Thus, it allows clustering patients who show similar activation of their factor sets (Figure 2).

## Results

### Sample Description

The sample involved 2254 patients, including 1184 (52.53%) women, 795 (35.27%) men, and 275 (12.20%) patients with missing data on sex. The mean age was 52.0 years (SD 15.1). Medical reports about the patients showed a CGI mean score of 2.95 (SD 1.95) (Multimedia Appendix 5) with a high percentage of participants scoring 3 (mildly ill; 844/2000, 42.20%) or 4 (moderately ill; 632/2000, 31.60%) and only a few scoring 6 or 7 (severely ill or extremely ill; 10/2000, 0.50%). According to the ICD-10 criteria, participants with mood disorders (F3; 347/1787, 19.43%), stress-related disorders (F4; 962/1787, 53.82%), and adult personality disorders (F6; 178/1787, 9.96%) represented most of the sample (Multimedia Appendix 6).

### Data Classification

The SPFM latent model analysis found the following three components in the assessment: one *bias term* and two *factor sets*. Both the bias term and factor sets involved groups of items of the questionnaire that are particularly informative. The bias term is present for all patients and reflects a common behavioral pattern. On the other hand, factor sets 1 and 2 are based on subsets of answers with high informational value to discriminate patients. Factor sets 1 and 2 can be present or absent for a particular patient. They are present if the corresponding subset of responses has a high score (value “1”: highest 10% scores) and are absent if the corresponding subset of responses has a low score (value “0”: remaining 90% of the scores).

The K-means algorithm applied on the Z matrix established four different patient profiles according to the presence of none, one, or both factor sets. Profile 0 presents only the bias term, profile 1 presents the bias term plus factor set 1, profile 2 presents the bias term plus factor set 2, and profile 3 presents the bias term and each factor set. The number of patients in each profile is shown in Table 1. In Table 2 and Multimedia Appendix 7, we can appreciate how each profile responds differently to the questionnaire.

**Table 1:** Number of patients in each profile.

Profile	Number of patients	Factor set
0	1113	Bias term (0)
1	480	0+1
2	616	0+2
3	45	0+1+2

**Table 2:** Average score for each item in the self-reported questionnaire of current mental well-being according to the β matrix of factor sets.

Questions	Profile 0 Bias term (0)	Profile 1 Factor set 0+1	Profile 2 Factor set 0+2	Profile 3 Factor set 0+1+2
1. How many hours did you sleep today? (from 0 to 12)^a^	0.0689	0.1525	0.0041	0.1567
2. Quality of sleep^a^	0.0390	0.2089	0.0040	0.2129
3. Do you have appetite?^a^	0.0446	0.1980	0.0009	0.1990
4. Do you take your medication?^a^	0.0795	0.0566	0.0225	0.0791
5. I felt joyful and with good mood^a^	0.0133	0.3228	0.0006	0.3235
6. I felt peaceful and relaxed^a^	0.0223	0.3181	0.0012	0.3193
7. I felt active and robust^a^	0.0130	0.3811	0.0103	0.3915
8. I felt awake, fresh, and rested^a^	0.0496	0.3894	0.0041	0.3936
9. My daily life has many interesting things^a^	0.0199	0.4236	0.0000	0.4236
10. Have you been able to keep focus on the tasks you did?^a^	0.0276	0.3467	0.0013	0.3481
11. Have you felt that you have a useful role in life?^a^	0.0187	0.3756	0.0018	0.3774
12. Have you felt able to make decisions?^a^	0.0173	0.3529	0.0002	0.3531
13. Have you enjoyed regular activities from daily life?^a^	0.0100	0.3739	0.0016	0.3756
14. Have you felt able to cope with your issues?^a^	0.0149	0.3674	0.0016	0.3691
15. Do you feel reasonably happy taking into account the circumstances?^a^	0.0174	0.3591	0.0030	0.3622
16. Do you feel aggressiveness?	0.1745	0.0053	0.2362	0.2415
17. Do you have suicidal thoughts?	0.3177	0.0012	0.4573	0.4585
18. Have you had worries interfering with your sleep?	0.0422	0.0210	0.2931	0.3142
19. Have you felt constantly overwhelmed or tense?	0.0109	0.0006	0.3108	0.3115
20. Have you felt unable to overcome your troubles?	0.0060	0.0015	0.3005	0.3021
21. Have you felt unhappy or depressed?	0.0001	0.0037	0.4213	0.4251
22. Have you lost self-confidence?	0.0083	0.0071	0.4784	0.4855
23. Have you felt worthlessness?	0.1097	0.0010	0.6198	0.6208

^a^The scores from these items were inversed during data processing.

### Patient Profiles

The bias term was associated with high scores in suicide thoughts and aggressiveness (items 5 and 6), as well as feelings of worthlessness (item 23). All patients in our sample shared the features of the bias term, but about half of them (n=1141) also presented one or two different factor sets. Those presenting factor set 1 were included in profile 1, which was characterized by the absence of positive mood, low sleep quality, low energy, and feelings of loss of control (items 1-3 and 5-15). Patients presenting factor set 2 were included in profile 2, which was characterized by intense suicidal thoughts, aggressiveness, intense feelings of depression and worthlessness, low self-confidence, and worries interfering with sleep (items 16-23). All these characteristics were simultaneously active in patients with profile 3, who presented simultaneously with both factors. No statistical differences were found between the profiles regarding the distribution of age or sex (*F*_3_=1.391, *P*=.24 and χ^2^_3_=0.56, *P*=.90).

### Association Between Patient Profile and Medical Evaluation

After modeling the data, we compared CGI scores and clinical diagnoses between profiles. The results showed that the CGI scores were higher than the mean in profile 1 (3.2, SD 1.27), with the largest percentage of participants evaluated with a score of 4 (192/453, 42.4%). For profile 2, the CGI scores were lower than the mean (2.78, SD 1.28), with a high percentage of participants evaluated with a score of 3 (256/557, 45.9%). Results in profile 3 were not compared given the low number of patients.

Most diagnoses fell within the F4, F3, and F6 categories in each profile and in the total sample, corresponding with affective disorders, neurotic and stress-related disorders, and disorders of adult personality and behavior. The distribution of participants with profile 1 was similar for all the types of diagnoses. However, profile 2 seemed to be more frequent among patients with diagnoses of schizophrenia and psychological, behavioral, and emotional disorders with onset in childhood/adolescence (F2: 43/90, 48%; F8: 3/5, 60%; and F9: 14/38, 37%; Table 3).

**Table 3:** Distribution of patient profiles according to the main ICD-10 diagnostic categories for psychiatric disorders (F0-F9).

Category	Profile, n (%)			
	0	1	2	3
F0	7 (50.0)	3 (21.4)	2 (14.3)	2 (14.3)
F1	26 (45.6)	13 (22.8)	18 (31.6)	0 (0.0)
F2	37 (41.1)	10 (11.1)	43 (47.8)	0 (0.0)
F3	215 (49.6)	104 (24.0)	104 (24.0)	10 (2.3)
F4	614 (51.2)	269 (22.4)	290 (24.2)	26 (2.2)
F5	51 (48.1)	25 (23.6)	29 (27.4)	1 (0.9)
F6	109 (49.1)	58 (26.1)	51 (23.0)	4 (1.8)
F7	6 (66.6)	1 (11.1)	2 (22.2)	0 (0.0)
F8	2 (40.0)	0 (0.0)	3 (60.0)	0 (0.0)
F9	20 (52.6)	3 (7.9)	14 (36.8)	1 (2.6)

## Discussion

The data modeling approach we applied was able to discriminate four different profiles of patients based on the answers to a brief electronic questionnaire. All profiles shared a component associated with feelings of aggressiveness, worthlessness, and suicidal thoughts (bias term or profile 0), which seemed to be common among patients who used e-mental health tools [33], such as the MEmind Wellness Tracker.

Sex and age distributions showed very little variability across the profiles, facilitating comparisons between them. In addition to profile 0 (bias term, default pattern), three profiles where found based on the scores of different sets of questions. It is important to bear in mind that a factor set was classified as active only when the scores were in the top 10% of the corresponding items. For example, even if the item of low sleep quality is absent from profile 2, a patient with that profile could still have high scores in that item compared with the general population and thus have relatively low sleep quality.

Patients in profile 1 reported a lack of positive mood, low quality of sleep, low energy, feelings of loss of control, and difficulties to face problems. These symptoms could be reactive to life difficulties and partly due to a lack of coping skills. Patients in profile 2 presented high scores in depressive feelings, worries interfering with their sleep, feelings of being overwhelmed and unable to overcome troubles, low self-confidence, and feelings of worthlessness. This pattern seems to be related with a greater inward focus and depressive-like symptomatology. Interestingly, patients in profile 2 also reported the highest scores for suicidal thoughts and feelings of aggressiveness. Indeed, patients in profile 2 reported five of the 10 ICD-10 diagnostic criteria for a depressive episode, including disturbed sleep, depressive feelings, reduced self-confidence, ideas of worthlessness, and ideas of suicide [26]. Surprisingly, those in profile 2 were evaluated by their physicians as having a higher level of functionality (CGI) than those in profile 1, despite higher levels of suicidal thoughts, aggressiveness, and depression in profile 2. This points to discordances between the medical assessment and the self-reported momentary assessment. Finally, profile 3 involved a small group of patients with high scores in all the items of the questionnaire. They shared the features of profile 1 and profile 2, and reported the most severely affected psychological state in our sample (the highest levels of distress).

Our study suggests that the analysis of data from electronic self-assessments can discriminate profiles or clusters of patients sharing similar clinical characteristics. These features do not seem to overlap with usual clinical diagnoses, since no differences were found in the prevalence of previous psychiatric diagnoses between profiles. Most patients in each profile received diagnoses in F4 (anxiety disorders) and F3 (mood disorders) ICD-10 categories, which were numerically the most common diagnoses in the sample. However, diagnoses of disorders with an onset during childhood and adolescence (eg, F8 and F9) and schizophrenia (F2) were overrepresented in profile 2. Profile 3 was particularly overrepresented among the small group of patients with organic mental disorders (F0) in the sample, which could implicate a more complex disease course. Interestingly, in a previous paper, we found that the assessments made by clinicians did not correlate well with patients’ self-reports within 24 h of a clinical evaluation [34].

The presence of sporadic suicide thoughts can be relatively frequent in psychiatric patients, but eHealth apps could help to identify profiles with higher suicide risk, such as profile 2. Previous literature has suggested improvements in mood, well-being, anxiety, and self-awareness, as well as a higher adherence to treatments among users of eHealth apps [5,35-37]. Electronic assessment tools, such as the one used in our study, may support physicians to discriminate patients with high suicide risk in order to adjust their interventions.

Among the limitations of our study, we note the use of only baseline assessments and incomplete clinical information. The described profiles might not be reflective of eHealth users who continue to use the app regularly. Besides, our intention was not to map the participants onto Diagnostic and Statistical Manual of Mental Disorders (DSM) or ICD categories but rather to identify symptomatic profiles that are not necessarily reflected in psychiatric diagnoses. This study was designed to explore the utility of a new method to classify e-mental health users, and it needs to be completed with follow-up data. Nonetheless, once the SPFM is trained, it will be possible to analyze changes in patient profiles during continuous assessment with several time points. It will also be possible to link the electronic assessment with medical records. Our results could help to select the most performing questions according to mental disorders or patient profiles, which, in turn, could be used to create shorter and more efficient questionnaires. We can see in our study that the question about medication intake had very low informative value.

There are still many concerns regarding e-mental health that need to be addressed. One of the main concerns reported by both professionals and users is related to the privacy, ownership, and responsible use of medical information [38,39]. This is one of the major challenges that eHealth needs to address by means of privacy-preserving technologies [40]. Accessibility and difficulties to find reliable sources of medical information are also important concerns in the population, especially among older adults [41]. Medical professionals also have doubts about the capacity of online information to improve the knowledge of patients and have reported concerns regarding the capacity of telemedicine to enhance physician-patient bond [39]. All these concerns must be addressed in order to improve the acceptability and use of eHealth tools. A recent study suggested that there is still a low preference for the use of eHealth tools among the adult general population [17]. However, those who have already used eHealth apps usually feel confident to continue using them. Some studies have reported a sense of security and the existence of a relational bond between eHealth apps and patients with psychiatric diseases [42,43]. Our analyses show that machine learning can help to classify e-mental health users and provide clues for their diagnoses and, importantly, their needs in terms of treatment. If machine learning helps physicians to take clinical treatment decisions based on data, the social perception about available eHealth tools will certainly improve.

## Appendix

Multimedia Appendix 1 List of categories from the ICD-10 Classification of Mental and Behavioral Disorders.

Multimedia Appendix 2 Clinical global impression (CGI) scores: severity of illness.

Multimedia Appendix 3 Dichotomized processing of data from the questionnaire scores.

Multimedia Appendix 4 A binary Z matrix, presenting the number of active factor sets for each patient. Each line corresponds to a single patient. All patients present the bias term or factor set 0.

Multimedia Appendix 5 Number of patients by clinical global impression (CGI) scores.

Multimedia Appendix 6 Percentage of patients according to ICD-10 diagnoses.

Multimedia Appendix 7 Average scores for each item of the self-reported questionnaire of current mental well-being according to the gamma distribution. The model is based on a β matrix. A higher weight (β) of a factor set for an item is associated with a greater probability to find a high score in that item when that particular factor set is active.

## Abbreviations

CGI: clinical global impression
IBP: Indian Buffet Process
ICD: International Statistical Classification of Diseases and Related Health Problems
SPFM: Sparse Poisson Factorization Model
