# Supplementary material for: Psychiatric Profiles of eHealth Users Evaluated Using Data Mining Techniques: Cohort Study
Source: JMIR Ment Health. 2021 Jan 20;8(1):e17116. doi: 10.2196/17116 (PMC7857940; doi:10.2196/17116)
Supplement: Multimedia Appendix 1 [file mental_v8i1e17116_app1.docx]

**Appendix 1.** List of categories from the ICD-10 Classification of Mental and Behavioral Disorders.

| **ICD-10 Code** | **Definition** |
| --- | --- |
| **F0** | Organic, including symptomatic, mental disorders |
| **F1** | Mental and behavioral disorders due to psychoactive  substance use |
| **F2** | Schizophrenia, schizotypal and delusional disorders |
| **F3** | Mood [affective] disorders |
| **F4** | Neurotic, stress-related and somatoform disorders |
| **F5** | Behavioral syndromes associated with physiological disturbances and physical factors |
| **F6** | Disorders of adult personality and behavior |
| **F7** | Mental retardation |
| **F8** | Disorders of psychological development |
| **F9** | Behavioral and emotional disorders with onset usually occurring in childhood and adolescence |
