# Supplementary material for: Psychiatric Profiles of eHealth Users Evaluated Using Data Mining Techniques: Cohort Study
Source: JMIR Ment Health. 2021 Jan 20;8(1):e17116. doi: 10.2196/17116 (PMC7857940; doi:10.2196/17116)
Supplement: Multimedia Appendix 2 [file mental_v8i1e17116_app2.docx]

**Appendix 2A.** Clinical Global Impression (CGI) scores: severity of illness.

Considering your total clinical experience with this particular population, how mentally ill is the patient at this time?

0 = Not assessed

1 = Normal, not at all ill

2 = Borderline mentally ill

3 = Mildly ill

4 = Moderately ill

5 = Markedly ill

6 = Severely ill

7 = Among the most extremely ill patients
