# Supplementary material for: Psychiatric Profiles of eHealth Users Evaluated Using Data Mining Techniques: Cohort Study
Source: JMIR Ment Health. 2021 Jan 20;8(1):e17116. doi: 10.2196/17116 (PMC7857940; doi:10.2196/17116)
Supplement: Multimedia Appendix 3 [file mental_v8i1e17116_app3.docx]

**Appendix 3.** Dichotomized processing data from the questionnaire scores.

| **Item** | **Percentage of ‘1’ values** | **Threshold** |
| --- | --- | --- |
| 1 | 9.94 | .62 |
| 2 | 9.75 | .74 |
| 3 | 9.67 | .78 |
| 4 | 11.22 | .9 |
| 5 | 23.51 | .9 |
| 6 | 46.49 | .9 |
| 7 | 9.93 | .83 |
| 8 | 9.76 | .87 |
| 9 | 10.16 | .9 |
| 10 | 15.31 | .9 |
| 11 | 9.71 | .88 |
| 12 | 9.89 | .72 |
| 13 | 13.49 | .9 |
| 14 | 9.85 | .9 |
| 15 | 9.63 | .84 |
| 16 | 9.98 | .85 |
| 17 | 9.94 | .9 |
| 18 | 9.72 | .89 |
| 19 | 9.4 | .87 |
| 20 | 11.49 | .9 |
| 21 | 15.7 | .9 |
| 22 | 29.06 | .9 |
| 23 | 9.58 | .9 |
