# Supplementary material for: Psychiatric Profiles of eHealth Users Evaluated Using Data Mining Techniques: Cohort Study
Source: JMIR Ment Health. 2021 Jan 20;8(1):e17116. doi: 10.2196/17116 (PMC7857940; doi:10.2196/17116)

**Appendix 4.** A binary Z matrix, presenting the number of active factor sets for each patient. Each line corresponds to a single patient. All patients present the bias term or factor set 0.
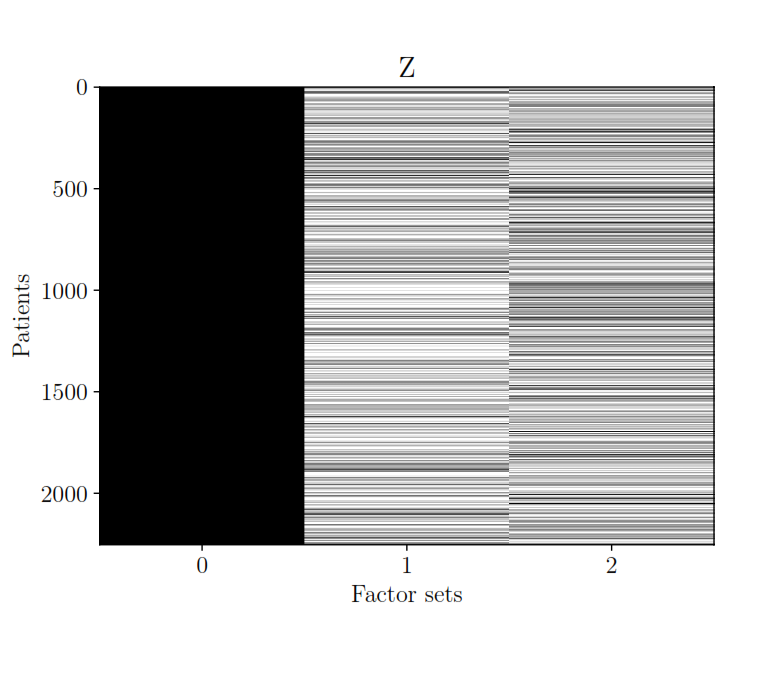

Supplement: Multimedia Appendix 4 [file mental_v8i1e17116_app4.docx]
