# Supplementary figures and images for: Psychiatric Profiles of eHealth Users Evaluated Using Data Mining Techniques: Cohort Study
Source: JMIR Ment Health. 2021 Jan 20;8(1):e17116. doi: 10.2196/17116 (PMC7857940; doi:10.2196/17116)

**Appendix 5.** Number of patients by CGI scores. Most patients scored 3 (mildly ill: 42.2%) or 4 (moderately ill: 31.6%).


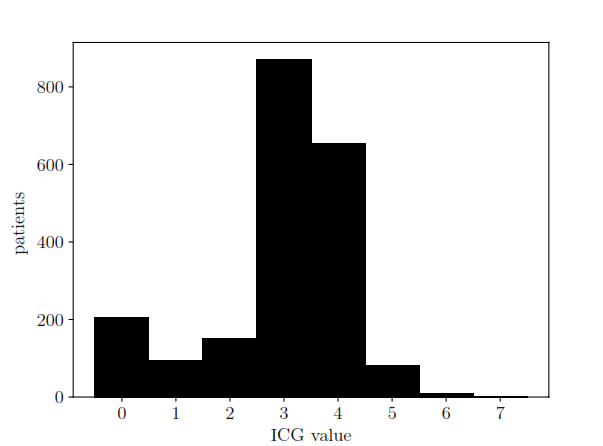

Supplement: Multimedia Appendix 5 [file mental_v8i1e17116_app5.docx]

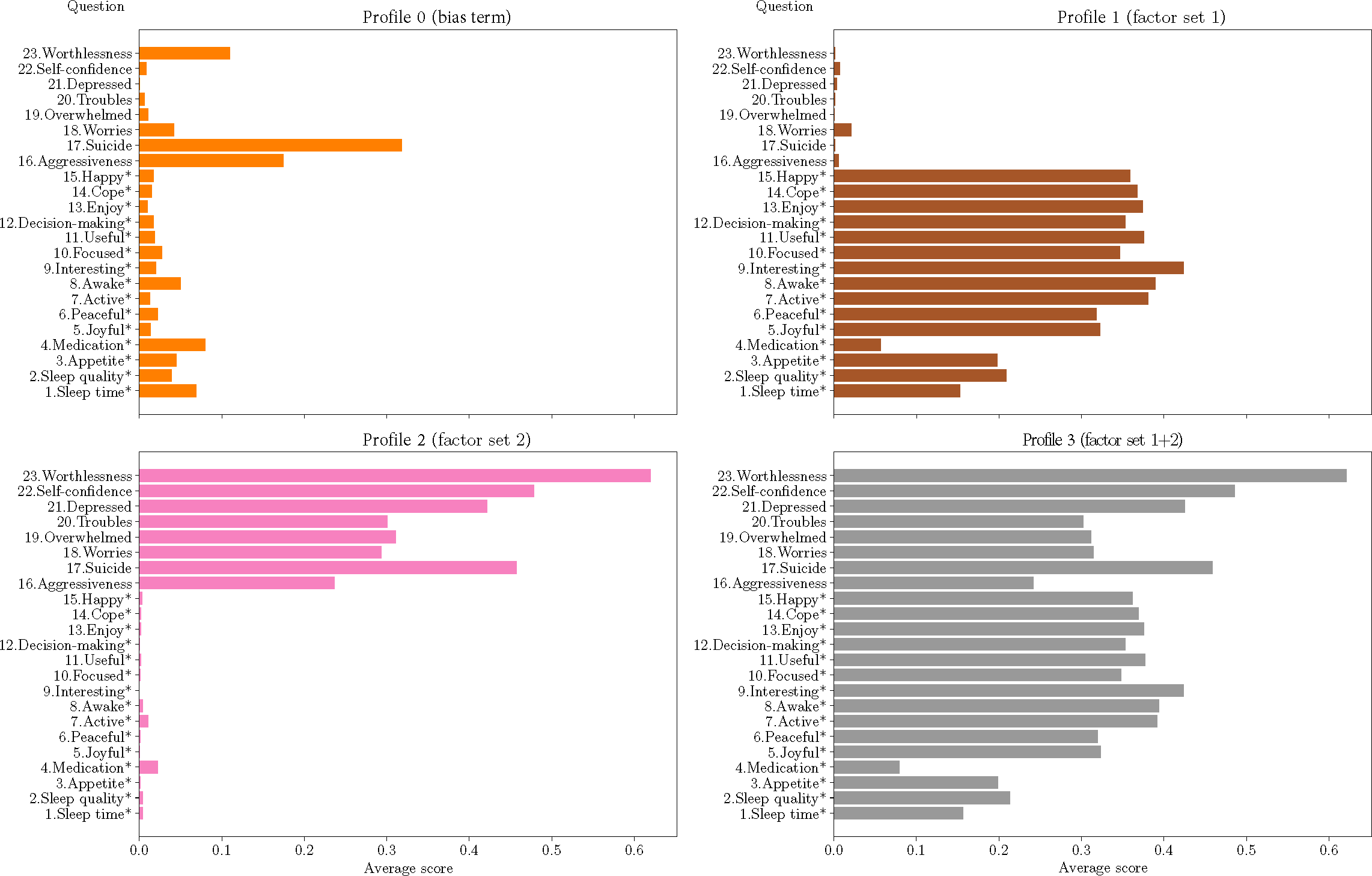

Supplement: Multimedia Appendix 7 [file mental_v8i1e17116_app7.png]
