# Supplementary material for: Psychiatric Profiles of eHealth Users Evaluated Using Data Mining Techniques: Cohort Study
Source: JMIR Ment Health. 2021 Jan 20;8(1):e17116. doi: 10.2196/17116 (PMC7857940; doi:10.2196/17116)

**Appendix 6.** Percentage of patients according to ICD-10 diagnoses. F0-F9 codes represent main ICD-10 diagnostic categories for psychiatric disorders. The most frequent disorders are in F3 (Mood disorders) and especially F4 (Neurotic, stress-related and somatoform disorders) categories.


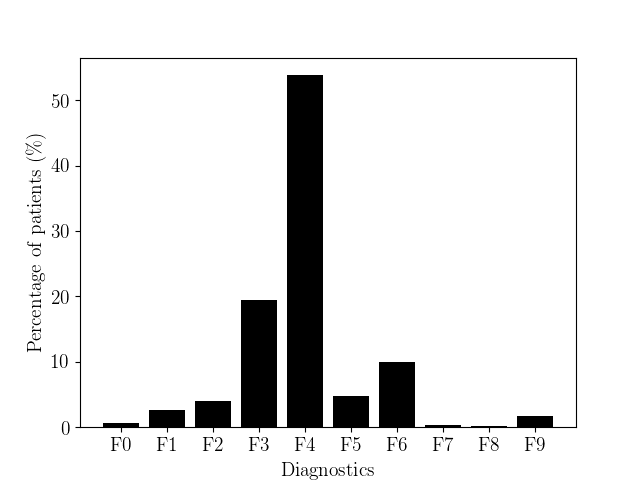

Supplement: Multimedia Appendix 6 [file mental_v8i1e17116_app6.docx]
